# Reaching the unreachable: a mixed-method evaluation of multidimensional healthcare model addressing the healthcare service gaps in hard-to-reach Northern riverine Bangladesh

**DOI:** 10.1186/s13690-025-01592-6

**Published:** 2025-04-14

**Authors:** Md Refat Uz Zaman Sajib, Kamrul Hasan, Tanvir Hayder, A M Rumayan Hasan, Md. Musfikur Rahman, Saraban Ether, Atia Rahman, Tania Sultana Tanwi, Fariya Rahman, Abu Sayeed, Sanwarul Bari, Syed Moshfiqur Rahman, Shams El Arifeen, Anisuddin Ahmed

**Affiliations:** 1 Department of Health and Kinesiology, University of Illinois Urbana- Champaign, Urbana, IL USA; 2 Maternal and Child Health Division, International Centre for Diarrhoeal Disease Research, Bangladesh (icddr,b), Dhaka, Bangladesh; 3 Strathclyde Law School, University of Strathclyde, Glasgow, UK; 4 Dalhousie University, Halifax, Canada; 5 Global Health and Migration Unit, Department of Women’s and Children’s Health, Uppsala University, Uppsala, Sweden

**Keywords:** 3-tier healthcare model, Comprehensive healthcare model, Hard-to-reach, Riverine low-resource setting, Bangladesh

## Abstract

**Background:**

Hard-to-reach riverine communities of northern Bangladesh face unique challenges in healthcare services. Friendship, an international social purpose organization, implemented a 3-tier healthcare model addressing these unique challenges over the past 20 years. This study evaluated Friendship’s 3-tier healthcare model in the northern riverine area, assessing service-seeking practices, experiences, stakeholders’ perceptions, and cost benefits for beneficiaries.

**Methods:**

A concurrent mixed-method approach was employed, including desk reviews, a cross-sectional quantitative survey, facility mapping, and qualitative interviews with service recipients, community representatives, healthcare providers, and health managers. Data were collected from five hard-to-reach riverine sub-districts across the Kurigram, Gaibandha, Bogura, Sirajganj, and Jamalpur districts of Bangladesh between April 2022 and July 2023. Data analysis followed major thematic domains for a comprehensive and complementary understanding.

**Results:**

A significant proportion (43.0%) of survey participants had no formal education, were aged 18–35 (57.5%), and earned less than 1,620 USD yearly (66.6%). Friendship’s healthcare services at the doorstep through satellite clinics and Female Community Medic Aides were widely accepted and preferred within the community for convenience, affordability (0.05–0.09 USD service charges), and superior quality, particularly the specialized treatments available on the hospital ships.

**Conclusion:**

Friendship’s 3-tier healthcare model made the accessibility and affordability of primary healthcare. Upon implementing a robust referral mechanism, continuing collaboration with the Government of Bangladesh, and expanding community awareness sessions to include topics such as mental health and disaster response, this model has the potential to be effective in similar settings in Bangladesh and other developing countries, as well as during emergency responses.

**Supplementary Information:**

The online version contains supplementary material available at 10.1186/s13690-025-01592-6.

**Table:** 

**Text box 1. Contributions to the literature**
• The study highlights how Friendship’s 3-tier healthcare model (hospital ships, monthly temporary paramedic-led clinics and 24/7 trained community medic aids) addresses existing challenges in access to healthcare services from government facilities in the hard-to-reach riverine areas of northern Bangladesh.
• This study examined the model’s role in promoting nutrition and healthy lifestyle practices, offering insights for scalable healthcare solutions in hard-to-reach communities.
• Such a multidimensional approach can also be cost-effective in providing essential healthcare services for hard-to-reach populations, instilling confidence in its sustainability and implication for similar settings.

## Background

In low-and middle-income countries (LMICs) like Bangladesh, the lack of healthcare resources and disparities in healthcare services are major challenges [1]. Such healthcare disparities impact the health indicators, delaying progress toward achieving Sustainable Development Goals (SDGs) [2]. Despite being a crucial component of development agendas, many developing countries continue to have significant concerns about the underutilization and limited reach of healthcare services [3].

Bangladesh, a densely populated country with a population of 164.7 million [4], faces significant challenges in meeting the rising healthcare needs of its diverse regions [5]. The persistent inequalities in healthcare access are mostly due to geographical diversity, including hard-to-reach areas such as coastal, hilly, haor (wetland), and riverine or Char areas (shallow land mass rising within a water body) [6, 7]. Despite constituting 5% of the total land with approximately 6.5 million people, these hard-to-reach areas generally lack adequate public health infrastructures [8]. Moreover, these Char areas are particularly vulnerable to natural calamities like flooding, erosion, cyclones, etc., which regularly hamper essential services, including healthcare and transportation. This further worsens the overall scenario, leaving the healthcare needs of such hard-to-reach Char communities mostly unmet. Previous studies have reported that only 35% of the Char dwellers have access to public healthcare services, while 80% are ultra-poor [8, 9]. As a result, seeking healthcare services from informal and traditional health practitioners remains frequent [9].

To aid the people of these underserved areas, Friendship, an international Social Purpose organization, has been working for the last 20 years through a multidimensional 3-tier healthcare model [10]. This includes Hospital Ships (Tier 1), Satellite Clinics and Static Clinics (Tier 2), and Friendship Community Medic Aides (FCMs) (Tier 3) [11–13]. Currently, Friendship operates two hospital ships: Lifebuoy Friendship Hospital (LFH) and Emirates Friendship Hospital (EFH), providing primary, secondary, and specialized healthcare in distant climate-vulnerable areas where the government and other non-governmental organizations have little or no intervention. Also, paramedic-led Satellite and Static Clinics serve the target community monthly to provide basic medical services for communicable and non-communicable disease prevention, behavioral change communication, and general healthcare services. Moreover, it trains female community members as medic aides (FCMs) to provide primary healthcare services in their communities [13].

Despite growing attention to improving access to healthcare among hard-to-reach populations, there have been few explorations conducted on the effectiveness [10] and long-term viability of community-based health approaches in riverine settings such as northern Bangladesh. Given the gaps in public healthcare coverage in these hard-to-reach areas, such a multidimensional model utilizing a mobility-based community participatory approach possesses potential efficacy [14]. Exploring this comprehensive healthcare model can reveal insights about how it functions in such a hard-to-reach setting and helps the community in need, as well as possible scopes to reinforce/introduce additional initiatives. Thus, this study aims to evaluate this 3-tier healthcare model addressing the healthcare needs of this hard-to-reach riverine area of northern Bangladesh in light of general healthcare service-seeking practices, experience, and perception from beneficiaries and stakeholders, as well as cost benefits.

## Methods

### Study design, duration, and site

This study adhered to a concurrent mixed-method approach integrating desk reviews, cross-sectional quantitative surveys, facility assessments, and qualitative design to comprehensively investigate the research objective. It was conducted between April 2022 and July 2023 in five northern riverine districts (Kurigram, Gaibandha, Bogura, Sirajganj, and Jamalpur) of Bangladesh, where Friendship runs the 3-tier multidimensional health model. Chilmari, Sundarganj, Sariakandi, Kazipur, and Madarganj sub-districts were selected randomly among the nine sub-districts, each representing one district.

Quantitative and qualitative data collection were conducted, focusing on the beneficiaries and stakeholders of this healthcare model. The cross-sectional survey was conducted to explore beneficiary satisfaction and quality of care regarding accessibility, service, cost, and perception of Friendship services and nearby facilities. Qualitative exploration was done alongside the quantitative survey to explore the knowledge and attitude of the community and stakeholders towards the model and evaluate the organisational efficiency and performance of the team for better correlation of different tiers (Hospital ships, Satellites, and FCMs). Also, the familiarity, level of trust, and perception of the community and stakeholders towards the healthcare model, including changes in the standard of living in the targeted communities, were explored through the qualitative approach. Moreover, observations by the field team during field and facility visits and field-site debriefing supported the qualitative exploration. Additionally, facility assessments in the Friendship hospital ships were done to evaluate the facilities’ available resources. To assess the financial cost-benefit from the beneficiary perspective and value for money from the provider perspective, a record review was conducted using administrative records from Friendship’s facilities and nearby private facilities. A financial cost-benefit analysis was executed from the beneficiary’s perspective, while value for money was assessed by estimating the direct cost of providing key services from the provider’s perspective.

### Sample size and sampling

The sample size for the quantitative survey was determined considering a 76% estimated prevalence (based on coverage) of beneficiaries in the targeted areas [15]. The margin of error and confidence level were assumed to be 4% and 99%, respectively. The total sample size for the survey was around 757 (~ 760) who had received healthcare services from this model at the study sites. The desired sample size for each sub-district was 152, which was distributed equally among the selected sub-districts.

To complement quantitative findings, approximately 30–36 qualitative interviews were planned to be conducted with purposively selected participants of different backgrounds (beneficiaries, service providers, administrative officials, and other stakeholders- community representatives/gatekeepers, local government officials, and health managers). However, 56 qualitative interviews were conducted following separate guidelines for different types of participants to reach data saturation. This included 32 In-depth interviews (IDIs) of the service recipients, providers, GoB health managers (UH&FPO, UFPO), and community representatives (local Union parishad chairman & member), ensuring maximum variation on saturation of information were carried out to explore the perception, knowledge, healthcare-seeking practice, level of trust, internal integration to carry the program. Moreover, 8 Focus group discussions (FGDs) with the service providers and service recipients were conducted to explore the perception of the healthcare model with a specific focus on its unique features like nutrition sessions, courtyard sessions, medicine distribution, etc. 16 Key-informant interviews (KIIs) were undertaken with national and local level GoB officials, program managers, Friendship’s local and national level officials, health managers, and healthcare providers to understand familiarity, level of trust, and perception of community and stakeholders.

Additionally, two facility assessments of the Friendship hospital ships and three case stories of the service recipients were conducted to support the findings comprehensively.

Finally, two review and feedback workshops were conducted, comprising GoB officials and health managers, service providers of different levels, and service recipients from different areas, to validate the collected data as well as seek recommendations for further integration and improvement. The findings were presented during the workshop, and feedback was taken from participants to ensure data validity.

### Data collection

Considering the population context and study location, the study recruited experienced data collectors: two males and two females. The selected data collectors received a week of rigorous training, including practical and supervised data collection sessions. The study team physically supervised field data collection to ensure high-quality data gathering. We used open-ended guides for KIIs, FGDs, IDIs, and structured questionnaires for the survey, which were finalized following initial field testing. The beneficiaries only residing permanently in the communities and have taken/have been taking services from the Friendship 3-tier healthcare model within/during the last one year were considered for the survey or interviews to understand the service-seeking behaviours, experience and perception of Friendship’s services. The KIIs were conducted face-to-face in convenient places for the respondents; FGDs and IDIs were held in the courtyards/households of the participants as they suggested. To ensure comfortable participation, no other people were allowed except the participants during the data collection process. For the survey, data were collected both from the satellite clinic and courtyard session participants and by visiting community households without separate scheduling rather than following regular satellite clinic schedules to different communities (venue-based data collection) [16]. Apart from these, observation notes of the field-site debriefing and review and feedback workshop were collected, and all previous reports and relevant records regarding each tier were conducted from Friendship regional and head offices as well as from the ships. Furthermore, for evaluating financial cost-benefit and value for money, data was collected through a record review from administrative records of Friendship’s facilities and nearby private facilities of the study sites. Data was collected for the five key services, such as the utilization of ANC, PNC, diarrhoea, skin disease, and respiratory tract infection, using a data collection tool for the cost of services. The service charges or costs of uptake of these services from nearby private facilities were also collected by physical visits from the visible service cost list or publicly available citizen charter from the respective organizations.

### Data analysis

Quantitative data (survey, reports, records, and logbooks) was stored in Excel and analyzed using STATA 15.0. Descriptive statistics were used to explain the findings and presented coherently with the qualitative findings. The cost of key services of Friendship’s 3-tier healthcare system was compared with nearby alternative facilities to calculate financial cost-benefit. For the value for money of the services from the organizational perspective, expenses to provide any specific services by Friendship (relative cost per beneficiary) were compared with nearby alternative private facilities. The service satisfaction level was measured using a quantitative questionnaire on a 5-point scale, which was further triangulated/validated through qualitative interviews. Moreover, the quantitative findings were narratively integrated with qualitative themes and systematically aligned to provide a side-by-side mixed-method interpretation.

Qualitative data (IDIs, KIIs, FGDs, workshops) were audio-recorded and analyzed thematically [17]. The qualitative data analysis was performed using NVivo software (Version 12) in several stages [18]. Initially, the purpose and plan of data analysis were outlined following listening to the tape-recorded interviews to identify discussed issues, emerging topics, and strengths and weaknesses of interview techniques. It helped to be familiarised with the data, enhanced future data quality, and initiated data analysis. The recorded interviews were then transcribed verbatim (conducted in the native language, Bengali), including all spoken content without alteration, and supplemented by field notes and interviewer’s observations. Subsequently, transcribed data were compared to identify data exploration gaps by assessing similar issues discussed by different types of interviewees. A set of a priori codes based on interview guidelines and study objectives were prepared before looking at the raw data and utilized to assign codes to each transcription. These codes were then condensed into a narrower set of codes and themes, ensuring comprehensive coverage of raw data. Next, emerging themes and sub-themes were identified, highlighting common ideas and recurrent themes grounded in actual data aligned with study objectives. Researchers implemented a systematic approach throughout the analysis process to ensure inter-coder reliability. This involved regular debriefings to discuss and examine the data collaboratively. The study reinforced the trustworthiness of its findings by engaging in thorough analysis and addressing both consensus and disagreement among researchers. To achieve scientific rigor, the researchers utilized triangulation, drawing from various data sources, including different participant categories, and employing multiple data collection techniques such as survey, facility assessment, KII, IDI, FGD, and observation. Additionally, data validation workshops and various analytical methods further contributed to the robustness of the results.

## Result

### Activities through the 3-tier healthcare model

**Fig. 1:**
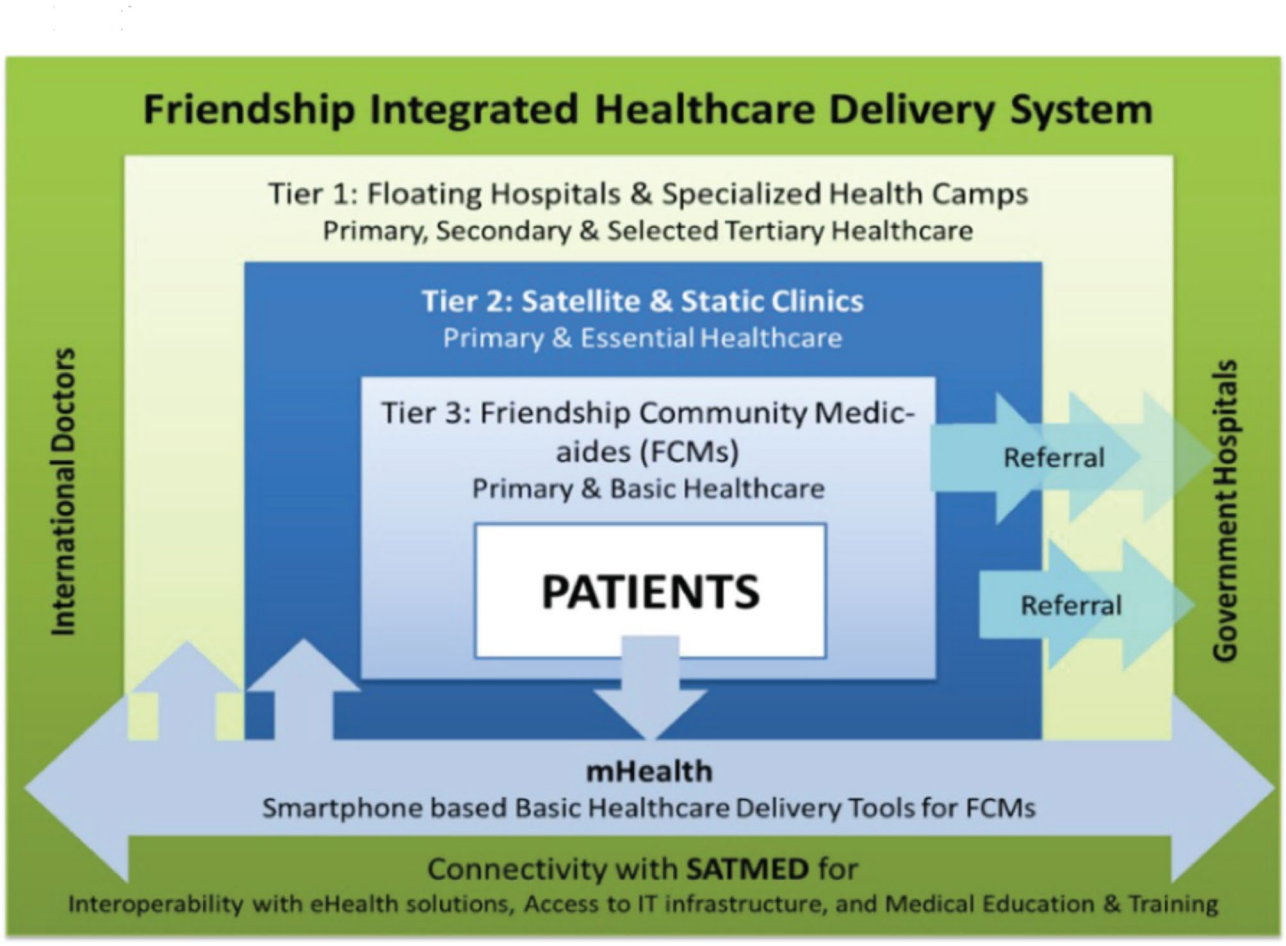
3-tier multidimensional healthcare model Source: Friendship Bangladesh

Friendship’s activities included various healthcare services but are not limited to antenatal care (ANC), postnatal care (PNC), family planning, respiratory tract infections, skin diseases, diarrhea, fever, cough, typhoid, and specialized services like cataract surgery, cleft lip and palate surgery, club foot surgery, etc. through the 3-tier healthcare model. It also involved monthly nutrition demonstration sessions, regular health and family care follow-ups (data tracking), assessment, treatment, and referral of malnutrition cases for under-5 children and pregnant and lactating mothers. Their hospital ships (Tier 1) provided primary, secondary, and specialized healthcare in distant climate-vulnerable areas where the government and other non-governmental organizations had little or no intervention, depending on the people’s needs and feasibility of moving, considering the navigability of the rivers. The paramedic-led Satellite and Static Clinics (Tier 2) run biweekly or monthly to provide basic medical services for communicable and non-communicable disease prevention, behavioral change communication, and general healthcare issues. Moreover, Friendship trained female community members as medic aides (FCMs) (Tier 3) to act as the first point of contact for Friendship healthcare initiative (Fig. 1). FCMs with specialized training were Community Skilled Birth Attendants (CSBAs) facilitating access to safe delivery, advanced family planning services, ANC, PNC, and basic infant/neonatal care for Char communities. Furthermore, some FCMs were equipped with mobile phones to enable m-health services for remote diagnosis and connecting patients with Friendship-assigned doctors when needed, following the app’s step-by-step instructions, ensuring comprehensive accessibility at any time.

### Sociodemographic and economic conditions

A significant proportion (43.0%) of the quantitative survey participants had no formal education. Among others, 26.8% attended primary school, 20.4% attended high school, and only 4.5% pursued college education. The majority of service recipients were female (88.9%) and aged 18–35 years (57.5%). Most households have 5—10 members (45.8%), approximately two-thirds (66.6%) earning less than 1620 USD, and 14.4% reported unawareness about their income. (Table 1).

**Table 1:** Sociodemographic and economic characteristics of survey respondents

Characteristics	*n* = 760 *N* (%)
**Sex**	
Male	84 (11.1%)
Female	676 (88.9%)
**Age category**	
18–25	210 (27.6%)
26–35	227 (29.9%)
36–45	111 (14.6%)
46–60	139 (18.3%)
61–90	34 (4.4%)
Don’t know	39 (5.1%)
**Household size category**	
1 to 4	406 (53.4%)
5 to 10	348 (45.8%)
> 10	6 (0.8%)
**Years of schooling**	
None	328 (43.1%)
1–5	204 (26.8%)
6–10	155 (20.4%)
10+	33 (4.5%)
Don’t know	40 (5.2%)
**Occupation**	
Housewife	585 (76.9%)
Auto/ Van/Rickshaw Driver	10 (1.3%)
Day Labour	12 (1.6%)
Fisherman	3 (0.4%)
Farmer	113 (14.9%)
Service Holder	8 (1.1%)
Small Business	18 (2.4%)
Others	11 (1.4%)
Yearly income (USD)*	
<=108	4 (0.5%)
108.01–540	96 (12.7%)
540.01–1080	217 (28.5%)
1080.01–1620	189 (24.9%)
1620.01–2160	80 (10.5%)
2160.01–2700	26 (3.4%)
>2700	39 (5.1%)
Don’t know	109 (14.4%)
Yearly expenditure (USD)*	
<=108	8 (1.1%)
108.01–540	172 (22.6%)
540.01–1080	256 (33.7%)
1080.01–1620	132 (17.4%)
1620.01–2160	46 (6.0%)
2160.01–2700	8 (1.1%)
>2700.01	4 (0.5%)
Don’t know	134 (17.6%)

*Converted using 01 BDT = 0.009 USD; OANDA currency converter as of 25 January 2023

According to the hospital ship register (2008 to 2022), for EFH and LFH, nearly 37–40% of beneficiaries were male, while around 59–62% were female, with over 70% of the beneficiaries aged between 26 and 60.

The overall findings are summarized in the Table 2. We organized them into four themes: health-seeking practices, experiences and perceptions, cost benefits, and challenges. In this table, we included both quantitative and qualitative findings, using mixed methods to interpret the data.

**Table 2:** Side-by-side presentation of mixed methods interpretations

Theme	Quantitative Findings	Qualitative Insights	Mixed methods interpretation
**General Health-seeking Practices**	90.8% used FCMs and satellite clinics; 12.8% accessed hospital ships for specialized care. 27.2% still relied on informal providers like quacks.	The community people were accustomed to obtaining services from Friendship due to easy access and quality services.	While quantitative data shows reliance on Friendship’s services, qualitative data reveals trust and perceived efficacy of the model’s offerings.
**Experience and Perception of the Service Recipients and Stakeholders**	83% of respondents were satisfied with the services; 82.4% appreciated the service environment at satellite clinics.	The respondents expressed satisfaction with organizing the courtyard session, collaborating with GoB, and specialized care.	Quantitative satisfaction levels are complemented by qualitative descriptions of community involvement and pride in the healthcare process.
**Cost Benefits**	Satellite clinic fees ranged from 0.05 to 0.09 USD, compared to 2.79 USD at private clinics. Transport costs to urban facilities were prohibitive (13.98–18.63 USD).	According to the participants, the community people received the services and medicine at a minimal cost without wasting extra time.	Cost analysis validates Friendship’s affordability, while qualitative accounts emphasize the importance of saving time and transport expenses.
**Challenges**	Absence of formal tracking systems for referred patients to govt. facilities or hospital ships	Heavy workload for service providers, as well as lack of training on skill development, indicated as challenges.	Both data streams underscore the need for a structured referral system to enhance continuity of care. Also, heavy workload and lack of training were highlighted in qualitative findings.

### General health Service-Seeking practices

**Fig. 2:**
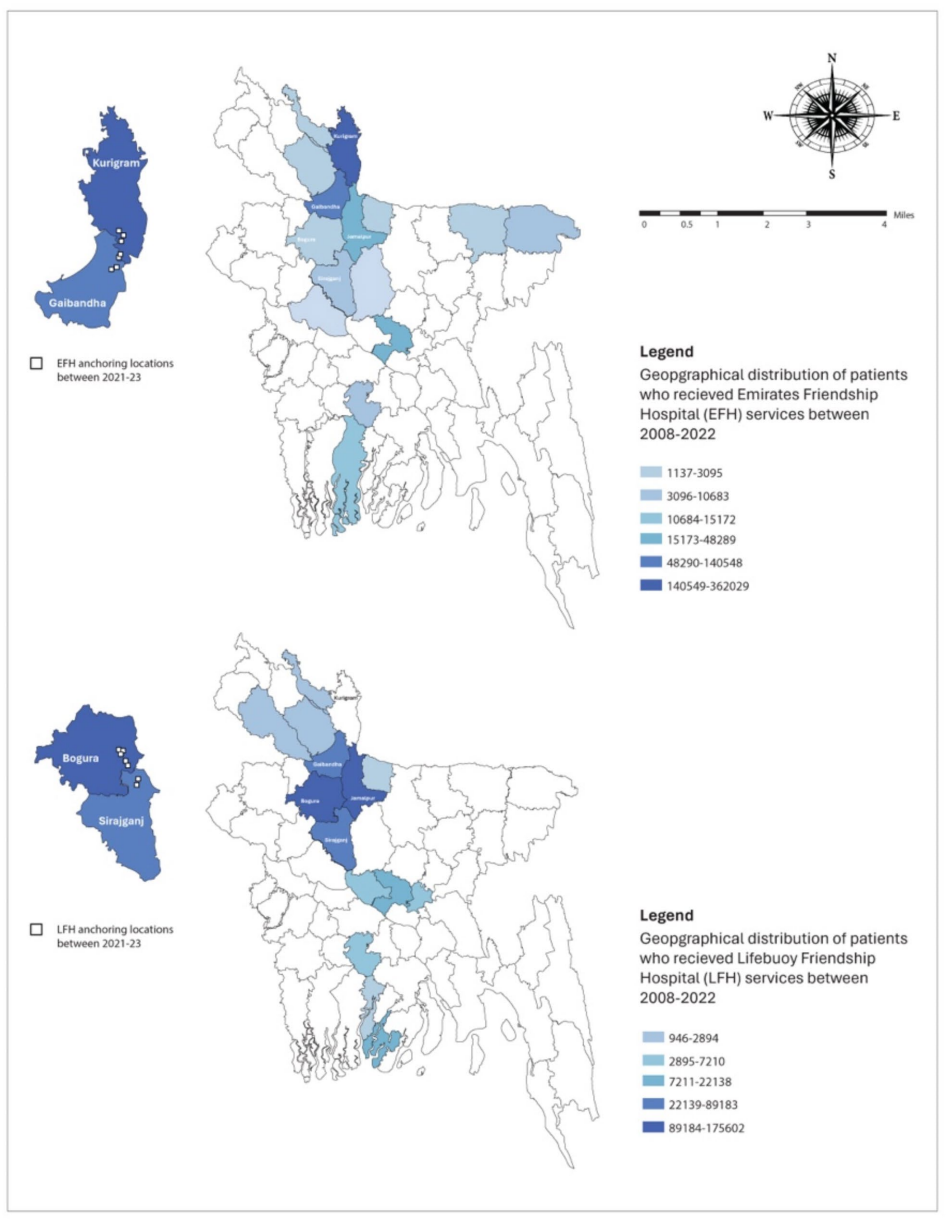
Geographical distribution of patients who received LFH and EFH services (2008–2022)

The Char people faced challenges in accessing government health services due to various factors such as geographical distance, transportation time, and cost associated with traveling to healthcare facilities. The 3-tier healthcare program provided convenient access to healthcare services in their neighborhood and enables them to get high-quality over-the-counter medicines at the most affordable price from the FCMs. The common health issues experienced by the residents of these areas included fever, common cold, diarrhea, dysentery, skin diseases, acidity, gastric pain, and worm infestation, among others. Over the last six months, 90.8% of the community people took healthcare services from the FCMs and Satellite Clinics, while 12.8% sought medical care from the hospital ships for common diseases. The hospital ship registers showed that approximately 551,289 and 647,090 individuals received services from the LFH and EFH, respectively, from 2008 to 2022. The geographical distribution of these beneficiaries (Fig. 2) indicates patients coming from distant locations beyond the ships’ primary catchment area. This could be attributed to high praise and positive word-of-mouth recommendations from patients and their relatives, as well as the availability of free and advanced specialized care provided by national and international doctors during these special camps.

Alongside Friendship services, they also received healthcare services from district hospitals (5.7%), Upazila Health Complexes (UHCs) (7.1%), Upazila Health and Family Welfare Centres (UH&FWCs) (0.7%), community clinics (1.6%), and community health workers (1.1%). Apart from these, 27.2% of people visited a village doctor/quack, while 19.8% purchased medicine based on recommendations from a drug seller/pharmacist. Furthermore, 6.2% sought treatment from a traditional healer. A 32-year-old female respondent (FGD) from the study area commented on FCMs services:

*“We can access the FCM at any time. It might be day or night*,* but we can call her for medical help. If she could not give us any suggestions*,* she contacted the MBBS doctor from Dhaka (capital of Bangladesh) over the phone.”*

The community people were familiar with and used to receiving services from the Friendship hospital ships, satellite clinics, and FCMs. The satellite clinic team typically sat up in the courtyard of FCM’s house to distribute medication and other services. The hospital ships provided routine basic and secondary healthcare services, including ANC, PNC, gynecology, pediatrics, family planning, obstetrics, etc. Friendship also organized specialized health camps where volunteer doctors, both local and international, performed surgeries and medical consultations. These health camps offered consultations for a range of health conditions, as well as provided specialized treatments like cataract surgery, cleft lip repair, and club foot correction. One of the respondents (IDI, male, 36y) stated:

*When the ship is accessible in this region, almost everyone prefers to visit the ship to reduce service expenses. We also receive quality services and medicines. Sometimes, they run camps for eye surgeries.*

### Experience and perception of the service recipients and stakeholders

Community people received specialized healthcare services from the Friendship hospital ships. In the past, people did not care much about their disabilities. Sometimes, they considered such disabilities as curses or “divine retribution.” Following the implementation of the 3-tier healthcare system, the population became well aware of cataract surgery, club foot treatment, cleft lip correction, etc. A participant (IDI, Male, 45y) from the study area stated:

*“We receive treatment for general health problems from satellite clinics. However*,* for more complex health issues*,* we are advised to visit the hospital ship. Once*,* I had a problem with my leg*,* and I was asked to visit the ship. There*,* I received treatment with check-ups*,* and it was very helpful for me. Also*,* I observed some people receiving spectacles after eye surgery there.”*

A service provider (KII, Male, 46y) stated that,

*“The health services of hospital ship play an important role in reducing many complex diseases such as eye*,* uterine*,* dental*,* club foot*,* general*,* etc. Now char-living people feel relieved about their health problems”.*

The community people consider the Friendship hospital ships to be a blessing. A female respondent (IDI, 58y) expressed her opinion:

*“Perhaps I couldn’t afford to go to a government or private hospital for my uterus problem; however*,* I received the operation at this ship with excellent care. The staff here carefully monitored me after the surgery.”*

At the satellite clinics, the “paramedic(s)” provided individualised care to the patients, ensuring appropriate privacy. They also prescribed the permitted over-the-counter medicines, which were distributed by the “organizer(s)”. If any medicines were not in stock on a given day, they advised the patients to buy them from nearby medicine stores. One of the service recipients (IDI, female, 43y) said,

*“The medicines we get from Friendship are better than what we buy from the local medicine shops. It worked*,* and we got better faster by taking the medicines from Friendship. But the types of medicines should be increased.”*

The majority of the community people expressed satisfaction with the services provided by the FCMs and Satellite clinics, mostly due to convenient access and proximity to their residences. Now, they don’t have to go on lengthy journeys to get medical advice and medicines. Overall, 83% of the community people were satisfied with the service, as well as, 82.4% were happy with the service environment of the satellite clinics. One of the recipients (IDI, female, 24y) shared the opinion:

*“We can avail healthcare in the courtyard of our house. We clean and arrange the courtyard where the clinic will take place ourselves. We don’t need to go to Sadar (urban centers) for minor illnesses anymore”.*

Apart from addressing the healthcare needs for different diseases, the nutrition sessions and the courtyard sessions created awareness and reinforced a nutrition drive. FCMs led the nutrition sessions, where they discussed the nutritional facts of locally produced vegetables and demonstrated how to cut and clean the vegetables while preserving their nutritional value. They also prepared nutritious and delicious “Khichuri,” a balanced diet, from locally sourced produce for the community children, as well as pregnant and lactating mothers. One of the community members (FGD, female, 35y) showed her satisfaction with the healthcare model and stated:

*“Friendship helps us to know the nutritional facts of vegetables. We had no idea that khichuri could be cooked using our locally available vegetables and be nutritious and delicious at the same time. Previously*,* we used to cook khichuri only with one type of lentil and no other vegetables or eggs. Now*,* we learned the whole procedure from the FCM Apa (sister)”.*

Courtyard sessions were usually conducted in a similar setting where FCMs discuss various topics such as diarrhea, respiratory tract infection, skin disease, hygiene, safe water and sanitation, food and nutrition, primary healthcare, gender and reproductive health, family planning, Extended Programme of Immunization (EPI) activities, pregnant mother care, labor planning, safe labor and PNC, care of newborn and benefits of breastfeeding, STIs and STDs, child marriage and adolescent reproductive healthcare, etc. using flip charts. One of the participants (FGD, female, 35y) shared,

*“We attend the session regularly and learned a lot from those sessions. For example*,* why we should wash our hands and when we must wash our hands. Our babies also adopted this behavior and washed their hands before eating. FCM apa also discussed birth control and family planning methods in the sessions”.*

A total of four courtyard sessions were conducted monthly in different locations throughout the community. Each session focused on health awareness issues and offered opportunities for social interaction and education on health and wellbeing. A participant (FGD, Male, 46y) mentioned:

*“Community people are now more aware about maintaining their health issues and healthy lifestyles. For example*,* pregnant women were not aware of their health during pregnancy before Friendship arrived here. But now they have learned many things*,* such as the five danger signs*,* and are aware of the four check-ups.”*

Friendship is the only Social Purpose Organization providing essential healthcare services in these hard-to-reach northern riverine Char communities. Though there were very few GoB Family Welfare Assistants (FWAs) in some districts (e.g., Jamalpur) to provide healthcare to the Char dwellers, the service was limited and insufficient considering the needs. One of the Upazila Health & Family Planning Officers (UH&FPO) (KII, Male, 42y) of the intervention area expressed his positive view and said,

*“By providing transportation support*,* Friendship made our work much easier. We have a very limited budget for transportation. Our EPI and Family Planning staffs use the boats of Friendship to go to the Char areas to implement and achieve the target set by the government. It’s possible because they maintain a strong liaison with us*,* with the government. Their staffs are very supportive. I would like to add that they work based on community people’s needs.”*

The strong collaboration in healthcare initiatives between Friendship and GoB reinforced the organization’s commitment to sustainability through public-private partnerships. Participants emphasized that continuous support underscored the government’s reliance on Friendship’s interventions to achieve national health goals, particularly in underserved areas. Another government health manager (KII, Male, 44y) mentioned,

*“We expect continuous support from Friendship to meet the government goals collaboratively. Even if there are any possibilities of discontinuation for any funding or other issues*,* please inform us beforehand. Otherwise*,* a lot of programme will be largely affected.”*

### Cost benefits

Satellite clinics charged flat fees of 0.05 USD for females and children aged under 18 years, and 0.09 USD for males. In the absence of these clinics, community people would need to seek alternative outdoor services at a private clinic with an average registration fee of 2.79 USD. The net benefit for a beneficiary using a specific service was 2.75 USD. Overall, 43.4% of the community people of the sample showed they were fully satisfied with the service charge of the satellite clinic, and 0.6% were fully dissatisfied, as recorded through the quantitative survey. Moreover, they had to endure lengthy and expensive journeys to reach the sub-district or district city. One of the participants mentioned (IDI, Male, 27y),

*Friendship team’s efforts in our community are commendable. We no longer have to waste 2–3 h travelling to reach the government Upazilla Health Complex for general illness. The best part is, they provide the services and medicine at a very minimal cost.*

The transport cost by reserved boat to the nearby port of other facilities ranged from 13.98 USD to 18.63 USD and it could take 2:00 to 2:30 h to reach the port. From there, it took a minimum of 30 min to 1 h to reach the healthcare facilities. There were also local or shared boats but with limited operating hours and routes from the Chars to lands. The costs of these boats ranged from 0.46 to 1.40 USD depending on the distance and geographical area.

Friendship’s five key services-antenatal care (ANC), postnatal care (PNC), diarrhea, respiratory tract infections, and skin diseases, were considered for cost-effective analysis. The relative cost per beneficiary for each service was − 0.96, -0.95, -1.02, − 0.74, and − 0.79 USD, respectively. The negative values indicate that the relative cost of providing the service by Friendship was lower than that of alternative private facilities.

### Challenges

Despite Friendship’s 3-tier healthcare model being well regarded for its cost-effective provision of essential healthcare services and higher satisfaction levels, there were still areas where improvements were crucial. The providers experienced a heavy workload and expressed the necessity for additional training in communication, counseling, management, and networking skills, as well as increasing the number of trained FCMS as CSBAs. Moreover, there was an immediate requirement to increase the number of providers, which was reflected in the quote from one of the project officers (KII, Male, 38y):

*We are always overwhelmed with lots of work compared to our salary. Also, we only receive the necessary training to perform our jobs. But it is crucial to get additional training to enhance our job performance and develop our skills. However, we do get verbal appreciation and motivation from our supervisors often.*

For the recipients, the primary barriers were long waiting periods to receive services at satellite clinics and limited mobility of the hospital ships due to poor navigability in dry seasons. Finally, despite providing support to the referred patients to government facilities or hospital ships for critical illness and specialized care, the absence of a formal and structured referral mechanism posed a significant challenge. Generally, patients were referred to Hospital Ships and government facilities (if hospital ships were not stationed nearby) for specialized care. However, there was a lack of systematic tracking of referred patients. This included insufficient follow-up and record keeping by FCMS or satellite clinics, as well as the lack of systematic tracking of referral status in hospital ships, even though the ship was considered the higher level of service point in this 3-tier model.

## Discussion

This evaluation study showed the utilization of Friendship’s 3-tier healthcare model to establish accessible healthcare services within the existing system in the hard-to-reach riverine areas of northern Bangladesh. Friendship’s 3-tier healthcare model presents similarities with a 3-tier healthcare system in rural China [19]. The Chinese 3-tier health service delivery was established to connect villages, townships, and counties and was implemented rapidly due to strong political commitment [20, 21]. Moreover, it was for the low and middle-income population, which is similar to the context of our study as explored by our quantitative survey- one-third of service users were of low-income status. In the Chinese 3-tier health service model, the village clinic was the primary institution for healthcare delivery. However, village clinics were unlikely to succeed due to shrinking healthcare resources and gaps, along with an absence of market mechanism interaction and administrative village mergers. Hence, Chinese VCs faced obstacles and concerns regarding access and engagement; however, FCMs from Friendship’s healthcare model made this easier due to having local people as FCMs as well as ample experiences and knowledge of the local populations [19].

Another review study demonstrated two-tier healthcare systems that involved transferring patients from a comprehensive hospital provider (CHP) to a primary hospital provider (PHP) and highlights the importance of effective coordination between the two providers to address the challenges of the referral system [22]. Although Friendship’s 3-tier healthcare model supports the patients seeking healthcare services at the government facilities (transportation, attendance and financial), it doesn’t have any formal referral mechanism between satellite clinics and hospital ships, the highest tier for specialized care within the system. Nevertheless, one study from Bangladesh revealed that rural people are often discouraged from visiting government hospitals due to their formal atmosphere and the unfriendly behaviour of healthcare staff, which leads to feelings of uncertainty and fear [23]. However, within the Friendship 3-tier healthcare model, as FCMs are females and from their own community, community members feel comfortable communicating with them as their first point of contact.

A study identified social, organizational, and physical barriers to accessing maternal healthcare services, including early marriage, perceptions of pregnancy and childbirth, high costs, lack of female health staff, inadequate health sector guidelines, distribution errors, low-quality services, and issues related to distance and waiting times [23]. According to our study, the Char people face similar challenges when obtaining healthcare from government and private healthcare facilities. But Friendship provided these services with easy access and low cost, maintaining the quality. Hence, the participants urged that government healthcare staff and other health services be made available in the Char communities.

Prior interventions in Bangladesh (Reach Up, Thinking Healthy, and general nutrition advice) created and piloted a culturally adapted integrated curriculum for pregnant and lactating women employing courtyard group sessions and household visits [24]. Friendship’s healthcare model incorporated nutrition sessions facilitated by FCMs to educate them on nutritious foods appropriate for their needs, as well as live demonstrations and distributions of nutritious ‘khichuri’ using locally available vegetables and eggs. These sessions possess high beneficence in promoting health awareness, as mentioned by the community people.

Another floating hospital initiative in Bangladesh, “Jibon Tari,” covers 27 locations in 18 districts, specially the riverine areas. Compared to Friendship’s Hospital Ships, the “Jibon Tari” provided general healthcare on prevention, cure, and awareness, addressing disabilities, as well as outdoor consultations and specialized surgeries for cataracts, club feet, post-polio deformities, loss of hearing, cleft lip, orthopedic, and ear, nose, and throat (ENT) [25]. However, the key distinction lies in portable clinic setup and permanent community representatives (FCMs) as well as implementing the 3-tier healthcare model coherently for comprehensive healthcare to the community. Moreover, Friendship healthcare system prioritised public-private collaboration, which contributes to the sustainability of such intervention, reflected by high praise from government officials.

In Indonesia, a similar remote situation persists due to territorial geography. There are 7,500 government health centers (puskesmas) along with floating hospital referral ships to improve public health services and address the challenge of unequal access to medical facilities [26]. Here, in the northern riverine areas of Bangladesh, the government and other health facilities were very limited. Friendship hospital ships, satellite clinics, and FCMs addressed these service gaps, increasing health literacy and awareness, and possessing high satisfaction among community people (83%). Thus, this comprehensive model has great potential to be utilised in other hard-to-reach areas (e.g., the southern coastal belt) in Bangladesh as well as in other similar low-resource settings. Moreover, during natural calamities or emergencies, the flexibility of the hospital ships can be highly effective in providing emergency healthcare services. Therefore, hospital ships can be a great addition to disaster preparedness programs in such climate-vulnerable regions like Bangladesh. Furthermore, the low-cost and mobile nature of the interventions eliminate the need for permanent infrastructure in the community, making it particularly suitable for riverine areas prone to erosion and frequent displacement of communities.

Transportation and cost hinder Char people from visiting sub-district and/or district hospitals. The reserved boats from the Char required 2:00 to 2:30 h to reach the nearby port and an additional 30–60 min to reach the healthcare facilities from there. A previous study on these populations revealed that 50% of the rich people travelled 30 min to receive services, while the corresponding percentage for the poorest was 23% [27]. However, through this 3-tier healthcare model, they received healthcare services in their community at minimal cost. With Friendship’s addressing transportation and cost barriers, the willingness to seek healthcare among Char people ultimately increased, as revealed in our exploration.

Moreover, this study explored the usage of integrated mHealth approaches along with regular 3-tier healthcare delivery, comparable to m-tika, an android-based solution for hard-to-reach areas ensuring full vaccination coverage for children in Bangladesh [28]. FCMs use mobile phones to connect with remote physicians, improving healthcare and overcoming the obstacles and challenges of overall Telemedicine services, as evidenced in previous studies [29].

One of the strengths of this study lies in its unique methodology. Due to the absence of prior data, the researchers relied on community members’ perceptions, revealing community opinions about their health management in rural areas as well as proxy indicators for the successful implementation of Friendship’s 3-tier healthcare model. Additionally, the relevant stakeholders, as well as government representatives, were included in the data collection process, providing a comprehensive view of the riverine population in Northern Bangladesh and revealing the actual state of affairs of health for the community people. Despite the best possible efforts, a limitation could be the potential inclusion of some non-permanent residents in some locations as beneficiaries due to riverine displacements. Moreover, the findings from this study may not be generalizable to general communities, considering the unique geographical and demographic characteristics of this region. Furthermore, due to a lack of sufficient information for the long intervention period, a complete economic evaluation was not feasible. Instead, a financial cost-benefit analysis for both providers and beneficiaries was carried out to provide a financial understanding. Finally, within the current study scope, the internal and external dynamics of the community could not be considered, which might have an impact on both health-seeking behaviors as well as healthcare delivery. Thus, future researches should consider such interplays of influencing social factors while evaluating healthcare models for hard-to-reach areas.

## Conclusion

For the hard-to-reach riverine areas in northern Bangladesh, Friendship’s multidimensional 3-tier healthcare model exhibits vital roles in terms of accessibility, lower cost, and improving healthcare service-seeking behavior. However, comprehensive training on emergency delivery procedures, mental health, and gender-based violence for the providers and implementing a digital inventory tracking and patient referral system are necessary to streamline the services and patient management. Additionally, efforts should focus on expanding community awareness sessions to include topics such as mental health, and disaster response. Furthermore, the coordination and cooperation with the government healthcare system can contribute to the sustainability of such interventions and be appreciated by the government and relevant stakeholders. Given its perceived effectiveness and efficiency from both beneficiaries’ and stakeholders’ sides, this multi-tier model has immense potential to be implemented in similar hard-to-reach riverine settings in other areas of Bangladesh as well as in other developing countries.

## Electronic supplementary material

Below is the link to the electronic supplementary material.

Supplementary Material 1

## Data Availability

The datasets used and/or analysed during the current study are available from the corresponding author upon request.

## Abbreviations

ANC: Antenatal care
CSBA: Community Skilled Birth Attendant
EFH: Emirates Friendship Hospital
EPI: Extended Programme of Immunization
FCMs: Friendship Community Medic Aides
FGD: Focus Group Discussion
FWA: Family Welfare Assistants
GoB: Government of Bangladesh
IDI: In-Depth Interview
KII: Key Informant Interview
LFH: Lifebuoy Friendship Hospital
LMICs: Low-and middle-income countries
PNC: Postnatal care
SDGs: Sustainable Development Goals
UH&FPO: Upazila Health and Family Planning Officer
UH&FWCs: Upazila Health and Family Welfare Centres
UHCs: Upazila Health Complexes
USD: United States Dollar
