# Supplementary material for: Reaching the unreachable: a mixed-method evaluation of multidimensional healthcare model addressing the healthcare service gaps in hard-to-reach Northern riverine Bangladesh
Source: Arch Public Health. 2025 Apr 14;83:103. doi: 10.1186/s13690-025-01592-6 (PMC11995601; doi:10.1186/s13690-025-01592-6)
Supplement: Supplementary file 1 — Supplementary Material 1 [file 13690_2025_1592_MOESM1_ESM.pdf]

Supplementary Materials: Study Tools

Title: Reaching the Unreachable: A mixed-method evaluation of multidimensional healthcare model addressing the healthcare service gaps in hard-to-reach Northern Riverine Bangladesh

Tool 1. Qualitative guidelines for FGD with Service Providers (Focus: Community Organizer, Paramedic)

1. **Job Responsibilities:** Please tell us details about your work responsibilities.
   a. Since when you started to work with *Friendship*?
   b. Describe about your area of work. E.g. no of household, name of the char, upazila, district.
   c. Mention the difference of responsibilities between m-health FCM and FCM?
2. **Service Details:** How/in which way you provide service in your intervention area? Please tell us about the procedure of getting service from you.
   a. Available health services provide
   b. No and type of medicines you can prescribe and provide?
   c. When you refer a patient and where do usually refer?
   d. How you make sure the patient is going to take the service from your referred place? Please tell about the referral system.
   e. Please mention about the follow up procedure of referred patient if you have any.
3. **Medicine collection & distribution process:** How do you collect medicine from *Friendship*? Please mention the whole process of medicine collection including the selling price and procedure to the community people.
4. **Service requirement & accessibility:** In what ways the interventions are meeting the needs of the service recipients and how essential these interventions in your community?
   a. E.g. service is improved and easily accessible than earlier? Reduced wait times while getting service? Help with self-management? Reduced travel time and expense?
   b. If community people face any difficulties to get service of *Friendship*'s intervention?
   c. Is the Friendship Health Model capable to provide effective coverage in reducing communicable and non-communicable disease burden?
5. **Community Feedback:** Have you elicited information from service recipient regarding their experiences with the intervention?
   a. What are their perceptions of the intervention? ·
   b. Can you describe what kind of specific information you have heard?
   c. Do you have any good experience that reflect the acceptability of users?
6. **Impact of Intervention:** How Friendship create difference in the life of Char areas community people by providing health care services of their intervention areas?
   a. Does people adopt positive health seeking behavior? Please compare the situation between the time of start and now, focusing the context of yours, Satellite Clinic and Floating Hospital Ship's service.
   b. Share any memory or incident that indicates the changes in health seeking behavior of community people.
   c. Does Friendship capable to provide effective coverage to reduce communicable and non-communicable diseases from the intervened community?
7. **Work-life Challenge:** What are the challenges are currently facing to implanting the activities in the intervention areas?
   a. Have you solved any of those?
   b. How did you solved please share such an event?
8. **Support & relation with supervisor/colleague:** Can you describe your working relationships with your colleagues and supervisor? With colleagues in your and/or other units?
   a. Do you meet (formally or informally) with your team? If yes, how often? Formally/informally?
9. **Staff Meetings and Interaction:** Does meeting held regularly?

   a. Agendas in the meeting
   b. No of staffs participates
   c. Frequency of meetings

10. **Staff Capacity Building:** Have you attended any training arranged by your organization?
    a. What was last training you get?
    b. How often training being conducted?
    c. Suggest topic of training you and field staffs need to give efficient service to the organization. What kinds of incentives are there to help ensure that the implementation of the intervention is successful?
11. **Motivation & Incentive:** What is your motivation for wanting to help ensure the implementation is successful?
    a. Have you get any appreciation or incentive from organization for your contribution?
    b. How's the relationship with your supervisor?
    c. To what extent do you think your supervisor will consider your role in this implementation in your (next) evaluation? In his/her regard for your work or role?
    d. Are there any special recognitions or rewards planned that are related to implementing the intervention? Can you describe them?
12. **Recommendation & Suggestion:** What kinds of changes or alterations do you think you will need to improve positive healthcare seeking behavior among the intervened community?

Tool 2. Qualitative guidelines for In-depth Interviews with Service Providers (Focus: Field Level Staff-FCM)

1. **Perception about Intervention:** Please describe about the services provide by *Friendship* in this intervention area. For instance; satellite clinic, nutrition program, cervical cancer survey, HH survey etc.
2. **Job Responsibilities:** Please tell us details about your work responsibilities.
    a. Since when you started to work with *Friendship*?
    b. Describe about your area of work. E.g. no of household, name of the char, upazila, district.
    c. mention the difference of responsibilities between m-health FCM and FCM?
3. **Service Details:** How/in which way you provide service in your intervention area? Please tell us about the procedure of getting service from you.
    a. Available health services provide
    b. No and type of medicines you can prescribe and provide?
    c. When you refer a patient and where do usually refer?
    d. How you make sure the patient is going to take the service from your referred place? Please tell about the referral system.
    e. Please mention about the follow up procedure of referred patient if you have any.
4. **Medicine collection & distribution process:** How do you collect medicine from *Friendship*? Please mention the whole process of medicine collection including the selling price and procedure to the community people.
5. **Service requirement & accessibility:** In what ways the interventions are meeting the needs of the service recipients and how essential these interventions in your community?
    a. Service is improved and easily accessible than earlier? Reduced wait times while getting service? Help with self-management? Reduced travel time and expense?
    b. If community people face any difficulties to get service of *Friendship*'s intervention?
    c. Is the Friendship Health Model capable to provide effective coverage in reducing communicable and non-communicable disease burden?
6. **Community Feedback:** Have you elicited information from service recipient regarding their experiences with the intervention?
    a. What are their perceptions of the intervention; e.g. nutrition session, courtyard session, medicine distribution? ·
    b. Can you describe what kind of specific information you have heard?
    c. Do you have any good experience that reflect the acceptability of users?
7. **Impact of Intervention:** How Friendship create difference in the life of Char areas community people by providing health care services of their intervention areas?
    a. Does people adopt positive health seeking behavior? Please compare the situation between the time of start and now, focusing the context of yours, Satellite Clinic and Floating Hospital Ship's service.
    b. Share any memory or incident that indicates the changes in health seeking behavior of community people.
    c. Does Friendship capable to provide effective coverage to reduce communicable and non-communicable diseases from the intervened community?
8. **Work-life Challenge:** What are the challenges are currently facing to implanting the activities in the intervention areas?
    a. Have you solved any of those?
    b. How did you solved please share such an event?
9. **Comparison with other similar Program:** Are there any other similar existing programs in your setting? If yes, how does the intervention compare to Friendship's intervention?
    a. What advantages & disadvantages of that intervention over Friendship's program?
10. **Support & relation with supervisor/colleague:** Can you describe your working relationships with your colleagues and supervisor? With colleagues in your and/or other units; e.g. paramedic, hospital ship, doctors at head office?

   a. Do you meet (formally or informally) with your team? If yes, how often? Formally/informally?

11. **Staff Meetings and Interaction:** Does meeting held regularly?
    a. Agendas in the meeting
    b. No of staffs participates
    c. Frequency of meetings
12. **Staff Capacity Building:** Have you attended any training arranged by your organization?
    a. What was last training you get?
    b. How often training being conducted?
    c. Suggest topic of training you and field staffs need to give efficient service to the organization. What kinds of incentives are there to help ensure that the implementation of the intervention is successful?
13. **Motivation & Incentive:** What is your motivation for wanting to help ensure the implementation is successful?
    a. Have you get any appreciation or incentive from organization for your contribution?
    b. To what extent do you think your supervisor will consider your role in this implementation in your (next) evaluation? In his/her regard for your work or role?
    c. Are there any special recognitions or rewards planned that are related to implementing the intervention? Can you describe them?
14. **Relationship with Local Community Leader:** Can you describe your working relationship with influential stakeholders? Have they discussed (formally/informally) anything about the intervention? If yes, can you explain about this?
15. **Recommendation & Suggestion:** What kinds of changes or alterations do you think you will need to improve positive healthcare seeking behaviour among the intervened community?

Tool 3. Qualitative guideline for in-depth interview for service recipients

1. **Familiarity with *Friendship*:** Do you know about *Friendship*?
   a. Since when *Friendship* started to work in this area? Please mention approximate time with the name of area, upazila and district
   b. What do you know about their activities?
   c. How do you know about their nutrition sessions, courtyard sessions and medicine distribution by *Friendship*?
2. **Requirement of service:** How essential these interventions in your community?
   a. Is there any other similar type health service provider providing service? If yes, then please describe those.
   b. In what ways the interventions are meeting the needs of health service of community people?
3. **Service availability:** Did you take any health service from *Friendship*?
   a. What type of service *Friendship* provides?
   b. Which service you took from them?
   c. What are the medicine available at satellite clinic and FCM?
   d. How to take the service from FCM, satellite clinic and hospital ship? Please tell details about the procedure.
   e. Besides *Friendship's* FCM and satellite clinic and/or hospital ship, where people usually go to take service?
4. **Service accessibility:** Can you easily get the service from FCM, satellite clinic/hospital ship?
   a. Usually how long does it take to avail service from satellite clinic? Please tell about the average time of getting service.
   b. Travel time and cost to take service?
5. **Impact of intervention:** How Friendship create difference in the life of Char areas community people by providing health care services of their intervention areas?
   a. Do you apply the health advises you get from FCM and satellite clinic in your daily life?
   b. Which advises you followed till now?
   c. Did you get any benefit by following those advises?
   d. Do you think you able to make changes in your life by taking health service and following health advises? Why or why not
   e. How it changes the standard of your living status?
6. **Service satisfaction:** How much you satisfied with the health service of *Friendship*? If yes/no, please explain why?
7. **Recommendation on service upgradation:** Please suggest, what kinds of changes or alterations do you think you will need to improve positive healthcare seeking behavior among the community people?

Tool 4. Qualitative guideline of focused group discussion for service recipients

1. **Familiarity with *Friendship*:** Do you know about *Friendship*?
   d. Since when *Friendship* started to work in this area? Please mention approximate time with the name of area, upazila and district
   e. What do you know about their activities?
   f. How do you know about their nutrition sessions, courtyard sessions and medicine distribution by *Friendship*?
2. **Requirement of service:** How essential these interventions in your community?
   c. Is there any other similar type health service provider providing service? If yes, then please describe those.
   d. In what ways the interventions are meeting the needs of health service of community people?
3. **Service availability:** Did you take any health service from *Friendship*?
   f. What type of service *Friendship* provides?
   g. Which service you took from them?
   h. What are the medicine available at satellite clinic and FCM?
   i. How to take the service from FCM, satellite clinic and hospital ship? Please tell details about the procedure.
   j. Besides *Friendship's* FCM and satellite clinic and/or hospital ship, where people usually go to take service?
4. **Service accessibility:** Can you easily get the service from FCM, satellite clinic/hospital ship?
   c. Usually how long does it take to avail service from satellite clinic? Please tell about the average time of getting service.
   d. Travel time and cost to take service?
5. **Impact of intervention:** How Friendship create difference in the life of Char areas community people by providing health care services of their intervention areas?
   f. Do you apply the health advises you get from FCM and satellite clinic in your daily life?
   g. Which advises you followed till now?
   h. Did you get any benefit by following those advises?
   i. Do you think you able to make changes in your life by taking health service and following health advises? Why or why not
   j. How it changes the standard of your living status?
6. **Service satisfaction:** How much you satisfied with the health service of *Friendship*? If yes/no, please explain why?
7. **Recommendation on service upgradation:** Please suggest, what kinds of changes or alterations do you think you will need to improve positive healthcare seeking behavior among the community people?

Tool 5. Qualitative guideline of In-depth Interviews with Community representative & local gatekeepers

1. **Familiarity with *Friendship*:** Do you know a NGO named *Friendship* is working in your area/upazila/district? Please share your ideas about the *Friendship*'s interventions in your area.
2. Is there any other organization currently operating health care services like *Friendship* doing? If so, please mention about their activities.
3. **Intervention Necessity:** How essential is this intervention of *Friendship* to meet the needs of the char living community people?
    a. To what extend *Friendship*'s three-tire health care model is being able to complement the existing government health service? Please share your opinion.
    b. In what ways will the intervention meet their needs? E.g. improved access to services? Reduced wait times? Help with self-management? Reduced travel time and expense?
    c. Do you think community people should be encouraged take more health service from *Friendship*?
    d. Which steps should be taken to encourage community people to using *Friendship* intervention?
4. **Impact of Intervention:** Do you think *Friendship* created difference in the life of Char areas community people by providing health care services? Please mention how, if possible share some examples.
    a. What is people's opinion about *Friendship*'s intervention?
    b. Does people adopt positive health seeking behavior due to their intervention? Please compare the situation between the time of start of intervention and now, focusing on the service currently available in your area.
    c. If possible, share any memory or incident that indicates the positive changes in health seeking behavior of community people.
    d. Does *Friendship* capable to provide effective coverage to reduce communicable and non-communicable diseases from the intervened community?
    e. What kinds of changes or alterations do you think will need more improvement to positive healthcare seeking behavior among the community?
    f. What factors will make the interventions more effective to change healthcare practice among Char living people?
5. **Communication & Collaboration:** Does Friendship maintain any communication/collaboration with government health program/personnel and local community leader/ influential stakeholder?
    a. Who are the key person maintaining liaison with government health service providers?
    b. How they maintain it, please mention.
    c. Do you think the Friendship's intervention is aligned in any magnitude to the government services? If yes, can you please explain how?
    d. Please suggest, how Friendship's current activities can be aligned more with the government services/initiatives?
6. **Feedback on intervention:** How do people feel about current programs/practices/process that are available?

Tool 6. Qualitative guideline of Key-Informant Interviews with Local & National Level Health manager

1. **Familiarity with *Friendship*:** Do you know a NGO named *Friendship* is working in your area/upazila/district? Please share your ideas about the *Friendship*'s interventions in your area.
2. Is there any other organization currently operating health care services like *Friendship* doing? If so, please mention about their activities.
3. **Intervention Necessity:** How essential is this intervention of *Friendship* to meet the needs of the char living community people?
    e. To what extend *Friendship*'s three-tire health care model is being able to complement the existing government health service? Please share your opinion.
    f. In what ways will the intervention meet their needs? E.g. improved access to services? Reduced wait times? Help with self-management? Reduced travel time and expense?
    g. Do you think community people should be encouraged take more health service from *Friendship*?
    h. Which steps should be taken to encourage community people to using *Friendship* intervention?
4. **Impact of Intervention:** Do you think *Friendship* created difference in the life of Char areas community people by providing health care services? Please mention how, if possible share some examples.
    g. What is people's opinion about Friendship's intervention?
    h. Does people adopt positive health seeking behavior due to their intervention? Please compare the situation between the time of start of intervention and now, focusing on the service currently available in your area.
    i. If possible, share any memory or incident that indicates the positive changes in health seeking behavior of community people.
    j. Does *Friendship* capable to provide effective coverage to reduce communicable and non-communicable diseases from the intervened community?
    k. What kinds of changes or alterations do you think will need more improvement to positive healthcare seeking behavior among the community?
    l. What factors will make the interventions more effective to change healthcare practice among Char living people?
5. **Association & Collaboration:** Does Friendship maintain any communication/collaboration with government health program/personnel and local community leader/ influential stakeholder?
    a. Who are the key person maintaining liaison with government health service providers?
    b. How they maintain it, please mention.
    c. Do you think the Friendship's intervention is aligned in any magnitude to the government services? If yes, can you please explain how?
    d. Please suggest, how Friendship's current activities can be aligned more with the government services/initiatives?
6. **Feedback on intervention:** How do people feel about current programs/practices/process that are available?

Tool 7. Guidelines for Key Informants Interview with Friendship Personnel (Focus- Friendship In-charge of District/Local Regional Office/Dhaka)

1. **Perception about Intervention:** What type of services are being provided by *Friendship* in the intervention areas through three-tire health care model? E.g. Nutrition Program, Satellite Clinic etc.
    a. Describe Nutrition program activities, cost of program conduct activities, no of children reach through nutrition program
    b. Satellite clinic activities, logistics taken to the field and type of services and medicines provide in clinic
2. **Intervention Area:** In which areas you are providing services through *Friendship*'s intervention? Please be Specific about the geographical areas;
    a. nature of the char areas (Land connection/only water body)
    b. accessibility with the main land
    c. no of chars including Char name, Upazila and District
3. **Service Details:** How many staffs are currently engaged in providing services? Please mention their position with their responsibilities.
    a. Type of health services according to their designation
    b. No and type of medicines they can prescribe and provide?
    c. mention the difference of responsibilities between m-health FCM and FCM?
    d. When they refer a patient and where they usually refer?
    e. How FCM make sure the patient is going to take the service from the referred place?
    f. Please mention about the follow up procedure of referred patient if you have any.
4. **Medicine collection & distribution process:** Please describe the whole procedure of purchasing medicines from the dealer to supply to the FCM?
    a. How FCM collects medicines from *Friendship*
5. **Transportation and connection with main land:** How your staffs go to the intervention areas? Please tell about the transportation they use to go to the intervention area.
6. **Duration of Service Providing Hour:** How long staffs stay and provide services in intervention area? Specifically, the hospital ships including satellite clinic staffs in char areas.
    a. No of patient they see during satellite as well as in hospital ship
7. **Service Accessibility:** How can community people get access to the *Friendship*'s health service. Please tell us about the procedure of getting service from FCM, Satellite Clinic and Floating Hospital Ship.
    a. Waiting time of service taking
    b. Travel time and expense while taking service
8. **Impact of Intervention:** How *Friendship* create difference in the life of Char areas community people by providing health care services of their intervention areas?
    a. What is people's opinion about Friendship's intervention?
    b. Does people adopt positive health seeking behavior? Please compare the situation between the time of start and now, focusing the context of FCM, Satellite Clinic and Floating Hospital Ship's service.
    c. Share any memory or incident that indicates the changes in health seeking behavior of community people.
    d. Does *Friendship* capable to provide effective coverage to reduce communicable and non-communicable diseases from the intervened community?
9. **Work-life Challenge:** What are the challenges are currently facing to implanting the activities in the intervention areas?
    a. Have you solved any of those?
    b. How did you solved please share such an event?
    c. Did you get support from your organization while facing challenge in this particular project?
    d. Also, compare the situation: now and then regarding the challenge faced

10. **Community Feedback:** Have you elicited information from service recipients about their experiences with the interventions of *Friendship*?
    a. Please mention the interval of time of data collection
    b. What type of data FCM collects?
    c. How the FCM collects data?
    d. Please share any incident that shows the satisfaction of the service recipients
11. **Reporting System:** Please share the reporting systems of the FCM, M-health FCM.
    a. Is there any specific format?
    b. Whom they usually report? Please describe the whole reporting channel.
12. Is there any other organization who are conducting similar activities in the health sector like *Friendship* doing? If so, what's their activities?
13. **Staff Meetings and Interaction:** Does meeting held regularly?
    a. Agendas in the meeting
    b. No of staffs participates
    c. Frequency of meetings
14. **Staff Capacity Building:** Have you attended any training arranged by your organization?
    a. What was last training you get?
    b. How often training being conducted?
    c. Suggest topic of training you and field staffs need to give efficient service to the organization.
15. **Association & Collaboration:** Can you describe your working relationship with influential stakeholders'/government health personnel?
    a. Have you discussed (formally/informally) anything about the intervention? If yes, can you explain about this?
    b. Does Friendship maintain any communication/collaboration with government health program/personnel and local community leader/ influential stakeholder?
    c. Who are the key person maintaining liaison with government health service providers as well as local community leader/influential stakeholder?
    d. How they maintain it, please mention.
16. **Financial Resource:** Do you always have sufficient financial support to implement the project activities properly?
    a. If yes, please describe the procedure of financial requisition and how you manage resource to conduct activities?
    b. Please describe what usually happen if you face financial resource constraints during project implementation.
17. **Recommendation & Suggestion:** Please give some suggestion to improve the interventions of this project.

Tool 8. In-Depth Interview (IDI) Guideline for Service Provider (Ship Assessment)

1. **Primary Idea on the three-tier healthcare model:** What is the three-tier healthcare model, and when and how did Friendship start providing health services through this model?
2. **Perception about floating ship hospital:** When did floating hospital start in Bangladesh? How many ships are providing the services in intervention areas?
    a. name of ships
    b. type of ship
    c. the existing facility in the hospital ship (bed, space, materials, etc.)

3. **Job Responsibilities:** Please tell us details about your work responsibilities.
    a. Since when you started to work with *Friendship*?
    b. Describe about your area of work.
    c. Mention the difference of responsibilities

4. **Intervention Area:** In which areas are you providing services through *Friendship*'s floating hospital? Please be specific about the geographical regions;
    d. nature of the char areas (Land connection/only water body)
    e. accessibility with the mainland
    f. no of chars including Char name, Upazila and District
5. **Target group or Beneficiaries**: Who takes health services from the floating ship hospital?
    a. Which type of people? (Rich, middle, or low-income)
    b. why are they receiving health services from here?

6. **Service Details:** What type of services are being provided by *Friendship* floating ship hospital? Describe the scope of the hospital ship's services in detail.
    a. The procedure for getting services
    b. Accommodation system
    c. Service cost
7. **Staff and Services:** How many staffs are currently engaged in providing services? Please mention their position with their responsibilities.
    a. Type of health services according to their designation
    b. No and variety of medicines they can prescribe and provide?
    c. Please mention the follow-up procedure of referred patient if you have any.
8. **Duration of Service Providing Hour:** How long staffs stay and provide services at floating hospitals?
    b. No of the patients they see in the hospital ship.
9. **Service Accessibility:** How can community people access *Friendship*'s health service in the floating hospital?
    c. Waiting time for service taking
    d. Travel time and expense while taking service
    e. Challenges to access for community people

10. **Medicine distribution process:** Please describe the whole procedure of medicines distribution. (probe: cost, difficulties, etc.)
11. **Transportation and connection with the mainland:** How community people go to the floating hospital? Please tell me about the transport they use to go to the ship.
12. **Impact of Intervention:** How *does Friendship create a difference in the lives* of Char areas community people by providing health care services in their intervention areas?
    e. What is people's opinion about Friendship's intervention?

f. Do people adopt positive health-seeking behavior? Please compare the situation between the time of start and now, focusing on the context of Floating Hospital Ship's service.
g. Share any memory or incident that indicates the changes in community health-seeking behavior.
h. Does *Friendship* capable of providing effective coverage to reduce communicable and non-communicable diseases from the intervened community?

13. **Work-life Challenge:** What challenges are currently facing in implanting the activities in the intervention areas?
    e. Have you solved any of those?
    f. How did you solve please share such an event?
    g. Did you get support from your organization while facing challenges in this particular project?
    h. Also, compare the situation: now and then regarding the challenge faced
14. **Community Feedback:** Have you elicited information from service recipients about their experiences with the Friendship interventions?
    e. Please mention the interval of time of data collection
    f. What was the data collection procedure?
    g. What was the result of the data collection?
    h. Please share any incident that shows the satisfaction of the service recipients.
15. **Reporting System:** Please share the reporting systems of the floating hospital.
    c. Is there any specific format?
    d. Whom do they usually report? Please describe the whole reporting channel.
16. Is there any other organization conducting similar activities in the health sector like Friendship? If so, what are their activities?
17. **Staff Meetings and Interaction:** Do meetings held regularly?
    d. Agendas in the meeting
    e. No of the staff participates
    f. Frequency of meetings
18. **Staff Capacity Building:** Have you attended any training arranged by your organization?
    d. What was the last training you got?
    e. How often is training being conducted?
    f. Suggest the topic of training you and the field staff need to give efficient service to the organization.
19. **Association & Collaboration:** Describe your relationship with influential stakeholders/government health personnel.
    e. Have you discussed (formally/informally) anything about the intervention? If yes, can you explain this?
    f. Does Friendship maintain communication/collaboration with government health programs/personnel and local community leaders/ influential stakeholders?
    g. Who are the key persons maintaining liaison with government health service providers as well as local community leaders/influential stakeholders?
    h. How they maintain it, please mention.
20. **Relationship with Local Community Leader:** Can you describe your working relationship with influential stakeholders? Have they discussed (formally/informally) anything about the intervention? If yes, can you explain about this?

21. **Financial Resource:** Do you always have sufficient financial support to implement project activities properly?
    c. If yes, please describe the procedure of financial requisition and how you manage resources to conduct activities.
    d. Please describe what usually happens if you face financial resource constraints during project implementation.
22. **Recommendation & Suggestion:** Please give some suggestions to improve this project's interventions.

Tool 9. In-Depth Interview (IDI) Guideline for service recipient (Ship Assessment)

1. **Familiarity with *Friendship*:** Do you know about *Friendship*?
   g. Since when *Friendship* started to work in this area? Please mention approximate time with the name of area, upazila and district
   h. What do you know about their activities?
   i. How do you know about their floating hospital and medicine distribution by *Friendship*?
2. **Requirement of service:** How essential these interventions in your community?
   e. Is there any other similar type health service provider providing service? If yes, then please describe those.
   f. In what ways the interventions are meeting the needs of health service of community people?
3. **Service availability:** Did you take any health service from *Friendship*?
   k. What type of service *Friendship* provides?
   l. Which service you took from them?
   m. What are the medicine available at ship hospital?
   n. How to take the service from floating hospital ship? Please tell details about the procedure.
   o. Besides *Friendship's* hospital ship, where people usually go to take service?
4. **Service accessibility:** Can you easily get the service from hospital ship?
   e. Usually how long does it take to avail service from hospital ship? Please tell about the average time of getting service.
   f. Travel time and cost to take service?
5. **Impact of intervention:** How Friendship create difference in the life of Char areas community people by providing health care services of their intervention areas?
   k. Did you get any benefit by receiving those services?
   l. Do you think you able to make changes in your life by taking health service and following health advises? Why or why not
   m. How it changes the standard of your living status?
6. **Service satisfaction:** How much you satisfied with the health service of *Friendship*? If yes/no, please explain why?
7. **Recommendation on service upgradation:** Please suggest, what kinds of changes or alterations do you think you will need to improve positive healthcare seeking behaviour among the community people?

Tool 10. Key Informant Interview (KII) Guideline (Ship Assessment)

Friendship Personnel (Focus- Friendship In-charge of District/Local Regional Office/Dhaka/Floating Hospital)

1. **Primary Idea on the three-tier healthcare model:** What is the three-tier healthcare model, and when and how did Friendship start providing health services through this model?
2. **Perception about floating ship hospital:** When did floating hospital start in Bangladesh? How many ships are providing the services in intervention areas?
   a. name of ships
   b. type of ship
   c. the existing facility in the hospital ship (bed, space, materials, etc.)
3. **Intervention Area:** In which areas are you providing services through *Friendship*'s floating hospital? Please be specific about the geographical regions;
   g. nature of the char areas (Land connection/only water body)
   h. accessibility with the mainland
   i. no of chars including Char name, Upazila and District
4. **Target group or Beneficiaries**: Who takes health services from the floating ship hospital?
   d. Which type of people? (Rich, middle, or low-income)
   e. why are they receiving health services from here?
5. **Service Details:** What type of services are being provided by *Friendship* floating ship hospital? Describe the scope of the hospital ship's services in detail.
   f. The procedure for getting services
   g. Accommodation system
   h. Service cost
6. **Staff and Services:** How many staffs are currently engaged in providing services? Please mention their position with their responsibilities.
   i. Type of health services according to their designation
   j. No and variety of medicines they can prescribe and provide?
   k. Please mention the follow-up procedure of referred patient if you have any.
7. **Duration of Service Providing Hour:** How long staffs stay and provide services at floating hospitals?
   c. No of the patients they see in the hospital ship.
8. **Service Accessibility:** How can community people access *Friendship*'s health service in the floating hospital?
   f. Waiting time for service taking
   g. Travel time and expense while taking service
   h. Challenges to access for community people
9. **Medicine distribution process:** Please describe the whole procedure of medicines distribution. (probe: cost, difficulties, etc.)
10. **Transportation and connection with the mainland:** How community people go to the floating hospital? Please tell me about the transport they use to go to the ship.
11. **Impact of Intervention:** How *does Friendship create a difference in the lives* of Char areas community people by providing health care services in their intervention areas?
    i. What is people's opinion about Friendship's intervention?
    j. Do people adopt positive health-seeking behavior? Please compare the situation between the time of start and now, focusing on the context of Floating Hospital Ship's service.
    k. Share any memory or incident that indicates the changes in community health-seeking behavior.

l. Does *Friendship* capable of providing effective coverage to reduce communicable and non-communicable diseases from the intervened community?

12. **Work-life Challenge:** What challenges are currently facing in implanting the activities in the intervention areas?
    i. Have you solved any of those?
    j. How did you solve please share such an event.
    k. Did you get support from your organization while facing challenges in this particular project?
    l. Also, compare the situation: now and then regarding the challenge faced
13. **Community Feedback:** Have you elicited information from service recipients about their experiences with the Friendship interventions?
    i. Please mention the interval of time of data collection
    j. What was the data collection procedure?
    k. What was the result of the data collection?
    l. Please share any incident that shows the satisfaction of the service recipients.
14. **Reporting System:** Please share the reporting systems of the floating hospital.
    e. Is there any specific format?
    f. Whom do they usually report? Please describe the whole reporting channel.
15. Is there any other organization conducting similar activities in the health sector like Friendship? If so, what are their activities?
16. **Staff Meetings and Interaction:** Do meetings held regularly?
    g. Agendas in the meeting
    h. No of the staff participates
    i. Frequency of meetings
17. **Staff Capacity Building:** Have you attended any training arranged by your organization?
    g. What was the last training you got?
    h. How often is training being conducted?
    i. Suggest the topic of training you and the field staff need to give efficient service to the organization.
18. **Association & Collaboration:** Describe your relationship with influential stakeholders/government health personnel.
    i. Have you discussed (formally/informally) anything about the intervention? If yes, can you explain this?
    j. Does Friendship maintain communication/collaboration with government health programs/personnel and local community leaders/ influential stakeholders?
    k. Who are the key persons maintaining liaison with government health service providers as well as local community leaders/influential stakeholders?
    l. How they maintain it, please mention.
19. **Financial Resource:** Do you always have sufficient financial support to implement project activities properly?
    e. If yes, please describe the procedure of financial requisition and how you manage resources to conduct activities.
    f. Please describe what usually happens if you face financial resource constraints during project implementation.
20. **Recommendation & Suggestion:** Please give some suggestions to improve this project's interventions.

Tool 11: Questionnaire for Cross-Sectional Survey

Section A: Demographic and socio-economic information					
**Q#**	**Question**	**Response**	**Code**	**Skip**	**Indicator**
A1	Respondent's name				
A2	Respondent's Area	Kurigram	1		
		Gaibandha	2		
		Bogura	3		
		Sirajganj	4		
		Jamalpur	5		
A3	Respondent's sex	Male	1		% of male/female/other participated in the study
		Female	2		
		Others	3		
A4	What is the date of birth of the respondent?	Month ______________<br>Year _________________			% of young (18-30 y)/young adult (31-40 y)/elder (≥41 y) participated in the study
		Don't know	999		
A5	Ask about the respondent's approximate age?	____________ Years			
		Don't know	999		
A6	What is the religion of the respondent?	Islam	1		% of Muslim/Hindu/Christian/Buddhist/others participated in the study
		Hindu	2		
		Christian	3		
		Buddhist	4		
		Other (specify)________________________	5		
A7	What is the highest educational qualification of the interviewer?	No education	0		% of participant with no education/incomplete primary/primary/below secondary or equivalent/secondary or higher
		Primary incomplete	1		
		Primary complete	2		
		Secondary incomplete	3		
		Secondary complete or higher	4		
		Don't know	999		
A8	What is the main occupation of the respondent?	Service Holder	1		# of Service Holder, Day Labour, Housewife, Farmer, Auto/Van/ Rickshaw Driver/Fisheries/Small Business, Others participated in the study
		Day Labor	2		
		Housewife	3		
		Farmer	4		
		Auto/ Van/Rickshaw Driver	5		
		Fisheries	6		
		Small Business	7		
		Other (specify)________________________	8		
A9	Number of family members of the respondent? (Count the respondent as well)	_______			Mean of family members of participants participated in the study
A10	What is the monthly expenditure of	_______________ Taka			

	the interviewer? (household/family income)	Don't know	999		Mean monthly expenditure of the participants

Section B: Access to Service and Service Seeking					
**Q#**	**Question**	**Response**	**Code**	**Skip**	**Indicator**
B1	Did you or your family members suffer from any diseases within the last 1 year?	Yes	1	→B3	
		No	2		
B2	Did you or your family members suffer from any diseases within the last 6 months?	Yes	1		
		No	2		
B3	If yes, what type of disease? (Multiple responses acceptable)	Fever and Cough	1		
		Pneumonia	2		
		Skin Diseases	3		
		Diarrhoea	4		
		Typhoid	5		
		Malaria	6		
		Jaundice	7		
		Other (specify) ____________________	8		
B4	Did you or your family members receive any treatment for the diseases?	Yes	1		
		No	2		
B5	If yes, where did you receive the treatment? (Multiple responses acceptable)	District Hospital	1		
		Upazila Health Complex	2		
		Union Health & Family Welfare Center	3		
		Community Clinic	4		
		Government Health Worker	5		
		Private Doctor/Clinic	6		
		Friendship Community Medic-Aide	7		
		Friendship Satellite Clinic	8		
		Friendship Floating Hospital	9		
		Village Doctor	10		

		Village Doctor	11		
		Pharmacy	12		
		Traditional Healer	13		
		Other (specify)___________________	14		
		Don't know	999		
B6	Where do you or your family members generally go for treatments? (Multiple responses acceptable)	District Hospital	1		
		Upazila Health Complex	2		
		Union Health & Family Welfare Center	3		
		Community Clinic	4		
		Government Health Worker	5		
		Private Doctor/Clinic	6		
		Friendship Community Medic-Aide (FCM)	7		
		Friendship Satellite Clinic	8		
		Friendship Floating Hospital	9		
		Village Doctor	10		
		Village Doctor	11		
		Pharmacy	12		
		Traditional Healer	13		
		Other (specify)___________________	14		
		Don't know	999		

Section C: Experience and Perception on the Services by Friendship Healthcare Model

Q#	Question	Response	Code	Skip	Indicator
C1	Did you or your family members take any services from the Friendship Health Model within the last 1 year?	Yes	1	→C3	% of participants took health care from Friendship Health Model in the last 1 year
		No	2		
C2	Did you or your family members take any services from the Friendship Health Model within the	Yes	1		% of participants took health care from Friendship Health Model in the last 6 months
		No	2		

	last 6 months?				
C3	If yes, then from which tier of the Friendship Healthcare Model? (Multiple responses acceptable)	Friendship Community Medic-Aide (FCM)	1		
		Friendship Satellite Clinic	2		
		Friendship Floating Hospital	3		
C4	If C1 yes, what kind of services? (Multiple responses acceptable)	Treatment of Fever and Cough	1		
		Treatment of Pneumonia	2		
		Treatment Skin Diseases	3		
		Treatment Diarrhoea	4		
		Treatment of Typhoid	5		
		Treatment of Malaria	6		
		Treatment Jaundice	7		
		Dental Care	8		
		Eye Care	9		
		Nutrition related Healthcare	10		
		Antenatal Care	11		
		Postnatal Care	12		
		Child Healthcare	13		
		Healthcare related to Breast and Cervix	14		
		Healthcare related to Sexually Transmitted Diseases	15		
		Healthcare related to Family Planning	16		
		Other (specify) ___________________________	17		
C5	Who advised you to take the services from the Friendship Healthcare Model? (Multiple responses acceptable)	Relatives	1		
		Neighbours	2		
		Friendship Community Medic-Aide	3		
		Not Applicable	4		
		Others (Specify________)	5		
C6	Do you know about the location of the Satellite Clinic of the Friendship Health Model?	Yes	1		
		No	2		
C7	Do you know about the Friendship Community Medic-Aide (FCM) of your area?	Yes	1		
		No	2		

C8	How much time did you wait to get the healthcare services from the Friendship Health Model?	0-10 minutes	1		
		10-20 minutes	2		
		20-30 minutes	3		
		above 30 minutes	4		
C9	Was the waiting time acceptable?	Yes	1		
		No	2		
C10	Regarding the environment of the services of the Friendship Health Model, your opinion-	Fully Satisfied	1		% of participants were satisfied with the environment of the healthcare services
		Satisfied	2		
		Neutral	3		
		Dissatisfied	4		
		Fully Dissatisfied	5		
C10	Regarding the behaviour of the healthcare provider of the Friendship Health Model, your opinion-	Fully Satisfied	1		
		Satisfied	2		
		Neutral	3		
		Dissatisfied	4		
		Fully Dissatisfied	5		
C11	Regarding the cost of the services of the Friendship Health Model, your opinion-	Fully Satisfied	1		
		Satisfied	2		
		Neutral	3		
		Dissatisfied	4		
		Fully Dissatisfied	5		
C12	Regarding the Friendship Health Model, your overall opinion-	Fully Satisfied	1		
		Satisfied	2		
		Neutral	3		
		Dissatisfied	4		
		Fully Dissatisfied	5		
C13	Will you recommend your friends and others to take services from the Friendship Health Model?	Yes	1		
		No	2		
		Neutral	3		
